# Development of a Mimotope Vaccine Targeting the *Staphylococcus aureus* Quorum Sensing Pathway

**DOI:** 10.1371/journal.pone.0111198

**Published:** 2014-11-07

**Authors:** John P. O’Rourke, Seth M. Daly, Kathleen D. Triplett, David Peabody, Bryce Chackerian, Pamela R. Hall

**Affiliations:** 1 Department of Molecular Genetics and Microbiology, University of New Mexico School of Medicine, Albuquerque, NM, United States of America; 2 Department of Pharmaceutical Sciences, University of New Mexico School of Medicine, Albuquerque, NM United States of America; New York State Dept. Health, United States of America

## Abstract

A major hurdle in vaccine development is the difficulty in identifying relevant target epitopes and then presenting them to the immune system in a context that mimics their native conformation. We have engineered novel virus-like-particle (VLP) technology that is able to display complex libraries of random peptide sequences on a surface-exposed loop in the coat protein without disruption of protein folding or VLP assembly. This technology allows us to use the same VLP particle for both affinity selection and immunization, integrating the power of epitope discovery and epitope mimicry of traditional phage display with the high immunogenicity of VLPs. Previously, we showed that using affinity selection with our VLP platform identifies linear epitopes of monoclonal antibodies and subsequent immunization generates the proper antibody response. To test if our technology could identify immunologic mimotopes, we used affinity selection on a monoclonal antibody (AP4-24H11) that recognizes the *Staphylococcus aureus* autoinducing peptide 4 (AIP4). AIP4 is a secreted eight amino acid, cyclized peptide produced from the *S. aureus* accessory gene regulator (*agr*IV) quorum-sensing operon. The *agr* system coordinates density dependent changes in gene expression, leading to the upregulation of a host of virulence factors, and passive transfer of AP4-24H11 protects against *S. aureus agr*IV-dependent pathogenicity. In this report, we identified a set of peptides displayed on VLPs that bound with high specificity to AP4-24H11. Importantly, similar to passive transfer with AP4-24H11, immunization with a subset of these VLPs protected against pathogenicity in a mouse model of *S. aureus* dermonecrosis. These data are proof of principle that by performing affinity selection on neutralizing antibodies, our VLP technology can identify peptide mimics of non-linear epitopes and that these mimotope based VLP vaccines provide protection against pathogens in relevant animal models.

## Introduction

The small particulate nature and multivalent structure of virus-like particles cause them to provoke strong immune responses and make them effective scaffolds for displaying heterologous antigens in a highly immunogenic format. Peptide-based vaccines are typically poorly immunogenic, however, peptides displayed on the surface of VLPs elicit high-titer and long-lasting antibody responses [1]–[5]. Although VLPs can be utilized to increase the immunogenicity of peptides, identifying relevant target epitopes and then presenting them to the immune system in a highly immunogenic context that mimics their native conformation, has largely been an unpredictable process of trial-and-error. The most widely used method for epitope identification is through affinity selection using peptide libraries displayed on a filamentous phage. This technology has identified the epitopes of many monoclonal antibodies (mAbs), and is a powerful technique for mapping linear epitopes and discovering peptide mimics of conformational and non-peptide epitopes. Nevertheless, peptides displayed on a filamentous phage are typically poorly immunogenic due to the low valency display of peptides on the phage surface. Thus, epitopes identified by phage display must be produced synthetically, linked to a carrier, and displayed in a structural context unrelated to the selected phage. Often, in this new conformation the peptides have vastly decreased affinity for the selecting molecule and frequently lose the ability to induce antibodies that mimic the selecting antibody.

VLP technology has not previously been adapted for use in epitope identification because recombinant VLPs are not well-suited for the construction of diverse peptide libraries. Insertion of heterologous peptides into viral structural proteins often result in protein folding and VLP assembly defects. [6]–[8]. To overcome these limitations, we engineered a version of the bacteriophage MS2 coat protein whose folding and assembly is highly tolerant of short peptide insertions [7]. This system has allowed us to generate large, complex libraries of VLPs displaying random peptide sequences. Because VLPs encapsidate the mRNA that encodes coat protein and its peptide [7], [9], VLPs with specific binding characteristics can be affinity selected and then the nucleic acid encoding the selected peptide can be recovered by RT-PCR. Most importantly, the same VLP can be used for both affinity selection and immunization. Thus, this system integrates the power of epitope/mimotope discovery of traditional phage display with the high immunogenicity of VLPs. We recently showed the utility of this VLP technology to identify linear epitopes and to elicit the proper antibody response by performing affinity selection using a set of well-characterized mAbs [10].

In this study we used this VLP vaccine discovery platform to identify immunogenic mimics of a quorum-sensing peptide from the Gram-positive pathogen *Staphylococcus aureus*. *S. aureus* is the leading cause of skin and soft tissue infections (SSTI) presenting to emergency departments in the USA [11]. The *S. aureus* accessory gene regulator (*agr*) quorum-sensing system coordinates a density dependent switch in gene expression that includes upregulation of virulence factors critical for invasive SSTI [12]–[15]. The *agr* system signals through the use of a secreted thiolactone-cyclized autoinducing peptide (AIP) which, upon binding to its cognate surface receptor AgrC, initiates a regulatory cascade leading to changes in transcription of more than 200 genes [16], [17]. Among the upregulated genes are those encoding secreted virulence factors essential for invasive skin infection, including upregulation of the pore-forming toxin alpha-hemolysin (Hla). Infection with *agr* or *hla* deletion mutants, loss of the Hla receptor ADAM10, or neutralization of Hla significantly attenuates virulence in mouse models of SSTI [13], [17]–[21]. Furthermore, we and others have shown that host innate effectors which disrupt *agr*-signaling also provide defense against *S. aureus* infection [22]–[26]. These results suggest that a VLP-based epitope identification approach to vaccine development targeted towards disruption of *agr* signaling would be efficacious against *S. aureus* SSTI.

Among *S. aureus* strains there are four *agr* alleles (*agr*I to *agr*IV) and strains from a given allele secrete a unique thiolactone cyclized AIP (AIPI to AIPIV) ranging from seven to nine amino acids in length. Due to their size, these peptides are inherently non-immunogenic. To overcome this, Park et al. described the production of a monoclonal antibody (AP4-24H11) against a synthetic AIP4 hapten that binds with nM affinity to AIP4 and that largely did not bind to other AIP family members, including AIP1 (µM affinity), which differs by a single amino acid [15], [27]. The crystal structure of AP4-24H11 bound to AIP4 reveals that the antibody recognizes the characteristic AIP thiolactone ring conformation, but does not interact with the N-terminal, linear region [28]. Importantly, passive transfer of AP4-24H11 protected against *S. aureus* pathogenicity in a mouse model of dermonecrosis and against a lethal intraperitoneal *S. aureus* challenge. The protection afforded by AP4-24H11 administration occurred without affecting normal bacterial growth, confirming that the AP4-24H11 mechanism of action was specific to inhibiting *S. aureus* virulence. Therefore, this work provided proof of principle that antibodies targeting AIP could be efficacious against *S. aureus* SSTIs [15].

We aimed to develop an active vaccine to provide protection against *S. aureus agr*-mediated pathogenesis. Traditionally, subunit vaccines utilize whole proteins, domains or epitopes conjugated to a carrier. We initially produced Qß VLPs with many copies of chemically conjugated AIP1 peptide, but they failed to elicit a protective response in the dermonecrosis mouse model (unpublished data). This failure may have resulted from potential instability of the native AIP molecule [15]. For example, the conformational restraint imposed by the AIP thiolactone bond is necessary for binding AgrC and induction of *agr*-signaling, as linearization of the AIPs results in loss of function. Furthermore, oxidation of the C-terminal methionine of AIP1 or AIP4 by host-generated reactive oxygen species is sufficient to inactivate the peptides. Thus, if the VLP-linked AIPs became linearized or oxidized during the conjugation or vaccination process, they would no longer be presented to the immune system as an authentic antigenic target. Therefore, we pursued the novel approach reported here using our affinity selection technology and the previously reported AP4-24H11 mAb targeting *S. aureus* AIP4.

Our approach was to identify peptides that immunologically mimic AIP4. Starting with random sequence peptide libraries on MS2 VLPs, we conducted biopanning on the AP4-24H11 mAb and identified 8 different VLPs displaying peptides that specifically bound the antibody. Vaccination with two of these VLPs elicited an immune response that protected in a *S. aureus* mouse model of dermonecrosis. These data demonstrate the feasibility of our VLP technology to identify immunologic peptide mimics of conformational epitopes and the potential to develop efficacious vaccines against otherwise non-immunogenic, conformationally constrained peptides such as those regulating *S. aureus agr*-dependent virulence. To our knowledge, this is the first report of an efficacious active vaccine targeting the secreted autoinducing peptides of the *S. aureus agr* quorum-sensing system.

## Materials and Methods

### Plasmid construction and random peptide libraries

The plasmids pDSP62 and pDSP62(am) were previously described [10]. Briefly, pDSP62 expresses the single chain dimer of the MS2 coat protein under the control of the inducible T7 promoter. VLPs produced using pDSP62 contain 90 copies of the displayed peptide per VLP. pDSP62(am) is the same construct except it contains an amber stop codon at the junction of the two coat protein monomers in the single chain dimer. The pDSP62(am) vector produces VLPs that display peptides at low valency (∼3 copies of the peptide per VLP) when expressed in a *E. coli* strain containing pNMsupA a plasmid that expresses an alanine-inserting, amber suppressing tRNA under the control of the lac promoter. The suppressor mediates occasional read through of the stop codon, so that pDSP62(am) produces a mixture of wild-type coat protein and the peptide-displaying single-chain dimer, which then coassemble into a mosaic VLP.

We have previously constructed random peptide plasmid libraries for use in our VLP affinity selection protocol that display peptides in the downstream AB loop of the MS2 single chain dimer coat protein [10]. Briefly, oligonucleotides were synthesized with 6, 7, 8 or 10 NNS codons, where N represents an equimolar mixture of all four nucleotides and S is an equal mixture of C and G. NNS codons encode all 20 amino acids and only a single stop codon. Using the Kunkel method, we produced plasmid libraries of at least 10^10^ individual transformants for each peptide library. All plasmids in this study were isolated using Qiagen Qiafilter or minipreps kits (Qiagen, Valencia CA).

### VLP production and purification

Plasmid libraries from affinity selection or single plasmids containing defined sequences that bound to AP4-24H11 were electroporated into the *E. coli*, T7 expression strain C41(DE3) (Lucigen, Middleton WI) and grown to mid-log phase. Coat protein expression was induced by the addition of IPTG (1 mM, Sigma-Aldrich, St. Louis MO) for three hours and bacteria were collected by centrifugation and the pellet was stored at –20°C overnight. Bacteria were lysed in SCB buffer (50 mM Tris, pH 7.5, 100 mM NaCl) by addition of 10 µg/ml of lysozyme, sonicated and purified from bacterial debris by centrifugation. The supernatant was treated with 10 units/mL of DNaseI (Sigma-Aldrich, St. Louis MO) and the VLPs were purified away from contaminating bacterial proteins by size exclusion chromatography using sepharose CL-4B resin (Sigma-Aldrich, St. Louis MO). Fractions that contained VLPs were combined and precipitated by the addition of ammonium sulfate at 50% saturation. Precipitated VLPs were collected by centrifugation, solubilized in SCB buffer and dialyzed in SCB overnight (Slide-a-lyzer cassettes 20 K MWCO, Millipore, Billerica MA). Purified VLPs were quantitated by Bradford assay (Biorad, Hercules CA) and analyzed by agarose gel electrophoresis and by SDS gel electrophoresis.

### Affinity selections

VLP affinity selection was performed on the neutralizing AIP4 monoclonal antibody AP4-24H11 (a generous gift from Gunnar Kaufmann and Kim Janda, Scripps Research Institute). VLP affinity selections were performed as previously described [10]. Briefly, for the initial round of selection, we coated Nunc MaxiSorp ELISA plates (eBiosciences, San Diego, CA) with 250 ng of AP4-24H11 in PBS overnight at 4°C. After washing, wells were blocked with 0.5% nonfat dry milk in PBS and the four VLP-peptide libraries (2.5 µg each of VLPs displaying 6, 7, 8 and 10 mers) were applied to the blocked wells for 2 hours (10 µg total VLP/well). After extensive washing (PBS), bound VLPs were eluted with 0.1 M glycine, pH 2.7 and immediately neutralized by the addition of 1/10 volume of 1 M Tris, pH 9. To make enriched VLP libraries for subsequent rounds of affinity selection, RNA from the eluted VLP were reverse transcribed and the RT products (containing the downstream coat protein and AB loop peptide) were amplified by PCR, digested with Bam HI and Sal I, and ligated into the pDSP62(am) vector. Ligation products were electroporated into the *E. coli* 10 G bacterial strain (Lucigen, Middleton WI), with a one-hour outgrowth and then immediately placed into 100 mL of LB media containing 60 µg/mL of kanamycin. After overnight growth, plasmids were isolated (Qiafilter Midi kit, Valencia CA) and used for VLP production for the next round of affinity selection. All plasmid libraries constructed after affinity selection contained at least 10^6^ individual transformants. Two additional rounds of affinity selection were performed with low valency peptide display (∼3 peptides/VLP); one using 250 ng of mAb per well and the final round used 50 ng of mAb per well.

### Identification and characterization of VLPs

After the final round of affinity selection, plasmid libraries enriched for VLPs displaying peptides that bound to the AIP4 mAb were isolated as described above. 1 pg of each library was electroporated into the C41(DE3) *E. coli* strain to ensure single transformants and bacteria were plated on agar plates containing kanamycin. The next day, single colonies were grown in 1 mL LB to an A_600_ of 0.6 and induced for VLP production with the addition of IPTG for 3 hours. Before induction, a 100 µL aliquot was removed for subsequent plasmid isolation. VLPs were isolated by sonication in SCB buffer and genomic DNA digested by incubation with 10 units of DNaseI. Crude VLP preps were assayed for binding to AP4-24H11 or an unconjugated control mouse IgG (Jackson ImmunoResearch Laboratories, West Grove PA) by ELISA. VLPs that bound to AP4-24H11 at least 5-fold higher then IgG control were further analyzed.

Plasmids were isolated from bacteria that produced VLPs that specifically bound to AP4-24H11, but not control antibody. Nucleotide sequences encoding the various peptides were determined (Eurofins Genomics, Huntsville AL) and plasmids encoding unique peptide sequences were electroporated into C41(DE3) for large scale VLP isolation as described above.

SDS PAGE and agarose gel electrophoresis was used to assess the purity and characterize the isolated VLPs. We ran 2 µg of total protein on a 10% NuPAGE SDS gel (Life Technologies, Grand Island NY) and stained the gel for total protein using Coomassie Blue (BioRad, Hercules CA). Since VLPs encapsulate their RNA we could also characterize VLP selectant particles by electrophoresis on an agarose gel. 10 µg of VLPs were run on a 1% TBE agarose gel containing ethidium bromide.

### ELISA

ELISA was used to assess relative binding of VLP selectants to AP4-24H11. Briefly, wells were coated with 250 ng of purified VLPs in PBS and incubated overnight at 4°C. Wells were washed 3 times with PBS and blocked for 1 hour using 3% BSA. Different concentrations of AP4-24H11 in 3% BSA were applied to each well, and incubated at room temperature for 1 hour. Unbound antibody was removed by washing with PBS. Goat anti-Mouse IgG HRP conjugated antibody (Jackson ImmunoResearch Laboratories, West Grove PA) was diluted 1∶5000 in 3% BSA and incubated for 1 hour at room temperature. ABTS solution (EMD Millipore, Billerica MA) was used to detect bound HRP antibody and color change was measured by absorbance at 405 nm (Opsys Plate Reader, Thermo Scientific, Waltham MA).

For competition ELISAs, plates were prepared as above. AP4-24H11 (100 ng/well) was mixed with different concentration of the cyclical, bioactive AIP4 peptide (10 µM–0.1 µM) for 10 minutes prior to incubation with VLPs. Secondary antibody and detection was the same as above. As control peptides, we used a linear form of AIP4 or a cyclical peptide from the L2 protein of human papillomavirus 16.

### Immunization and skin infection model

Animal studies were carried out in accordance with the recommendation in the Guide for the Care and Use of Laboratory Animals, the Animal Welfare Act, and U.S. federal law. The protocol was approved by the Institutional Animal Care and Use Committee (IACUC) of the University of New Mexico Health Sciences Center. Four to six week old female Balb/c mice (Harlan Laboratories, South Easton MA) were immunized with 10 µg of VLPs in PBS (50 µL total) without the addition of adjuvant by intramuscular injection into the caudal thigh muscle. The initial immunization was followed with 2 boosts each 2 weeks apart. The initial experiment used 3 mice for each VLP and subsequent experiments used 5 mice per vaccine candidate. As negative controls, 2 groups of mice were immunized with either PBS alone or a control VLP (displays no peptide).

The dermonecrosis model of mouse skin infection was previously described and performed with minor modifications [29]. Briefly, one week after the final vaccine boost, mice were anesthetized with isoflurane and inoculated subcutaneously with 1×10^8^ CFU of early-exponential phase *S. aureus agr*IV (AH1872–MN TG; generously provided by Dr. Alex Horswill, University of Iowa [30]). Animals were monitored for weight loss and lesion formation for three days post-infection. Lesion formation was assessed by measuring the maximal width and length of the abscess and necrotic ulcer with calipers. Area of the abscess was determined using the equation A = (π/2)×L×W, while the necrotic ulcer area was determined using the equation A = L×W [20]. On day three post-infection animals were euthanized by CO_2_ asphyxiation and abscesses (2.25 cm^2^ area) were collected. The tissue was homogenized and serially diluted for CFU enumeration.

### Cytokine analysis of abscess tissues

Abscess homogenates were stored at –80°C until cytokine analysis. Homogenates were rapidly defrosted at 37°C and clarified by centrifugation at 12,500×*g* for 10 minutes. Cytokine concentrations in clarified supernatants were determined using a custom designed multiplex assay performed as per manufacturer’s recommendations (EMD Millipore, Billerica, MA). The assay was read on a Bio-Plex 200 instrument and data analyzed using the Bio-Plex Manager Software (Bio-Rad, Hercules, CA). Statistical analysis of all in vitro data was performed using the two-tailed Student’s t-test.

### Alpha-hemolysin Western blot

Frozen homogenates were thawed and clarified as described above. Briefly, clarified tissue homogenate was separated by SDS-PAGE on a 16% Tris-glycine gel (Life Technologies, Grand Island, NY) before transfer to a polyvinylidene fluoride membrane. After blocking using TBST (20 mM Tris pH 7.5, 150 mM NaCl, 0.1% Tween 20) with 5% non-fat dry milk, immunodetection was performed using an anti-Staphylococcus alpha hemolysin antibody (Abcam, Cambridge, MA). Immunoreactive band intensity was determined using a FluorChem R System and AlphaView software (proteinsimple, Santa Clara, CA). Relative intensity is the ratio of measured intensity divided by the total protein concentration based on absorbance at 280 nm.

### Statistical analysis

Statistical significance was determined using GraphPad Prism v.5.04. The two-tailed Student’s *t*-test was used for analysis of in vitro data, and in vivo data were analyzed by the Mann-Whitney U test for non-parametrics. Results were considered significantly different at p<0.05.

## Results

In order to identify mimotopes of the AIP4 mAb AP4-24H11 epitope, we performed affinity selection on AP4-24H11 using our random sequence peptide libraries displayed on MS2 VLPs. The basic methodology is found in Figure S1. We used a mixture of four libraries, each displaying 6-, 7-, 8- or 10-amino acid inserts, with each library containing more than 10^10^ transformants [7], [10]. We find that many antibodies have strong preferences for peptide sequences of specific lengths, and using a mixture increases the probability of finding optimal binding peptides. Three iterative rounds of affinity selection were performed, each at increasing stringency; in round 2 we increased the stringency by reducing the display valency from 90 to about 3 peptides per particle. In round 3 stringency was further increased by reducing the amount of antibody 5-fold to 50 ng. Reaction of the selectant population with AP4-24H11 was monitored by ELISA after each round. By the end of round 3 binding was elevated more than 200-fold (data not shown).

**Figure 1:**
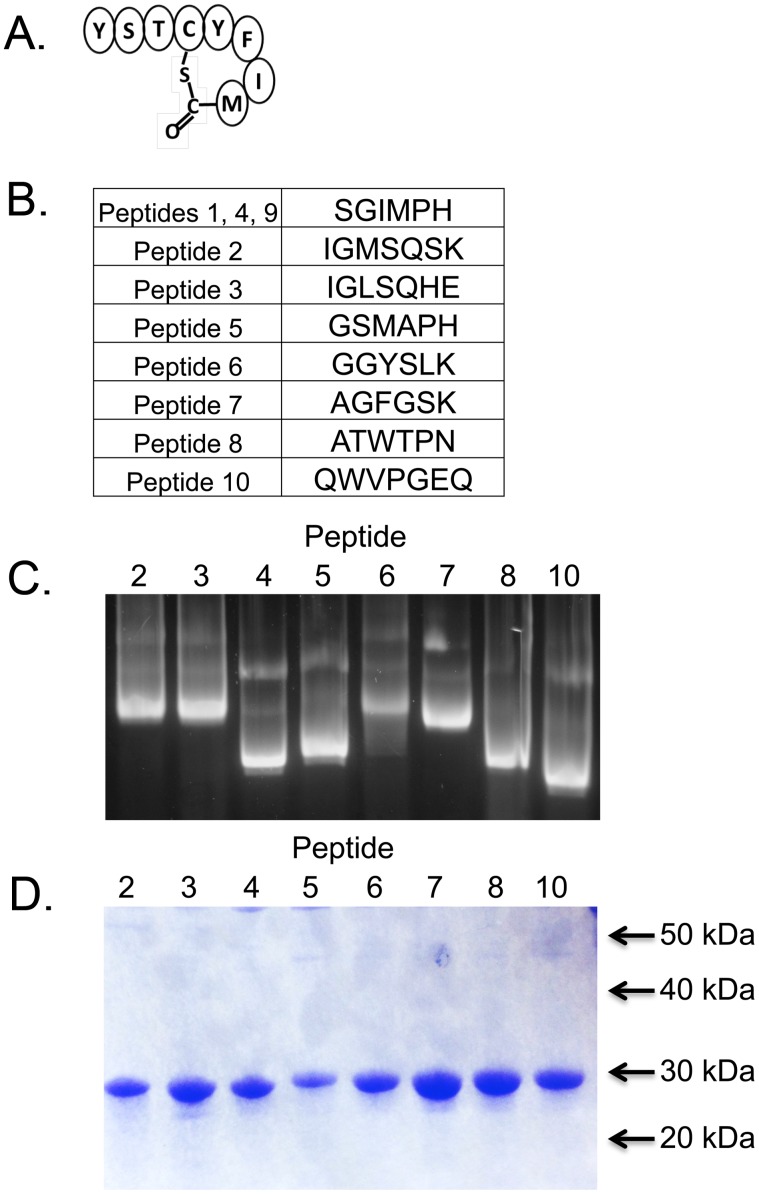
Identification and purification of affinity selected VLP displayed peptides. (A) The sequence and structure of the AIP4 peptide. (B) 10 VLP clones were sequenced after three rounds of VLP affinity selection. Peptide inserts are shown and demonstrate little primary sequence homology to the native AIP4 peptide. (C) Agarose gel analysis of purified VLPs. RNA staining is indicative of intact VLPs (which encapsulate their RNAs) and differences in VLP mobility are mostly due to differences in the charges of the peptides displayed on the VLP surface. (D) SDS-PAGE. Two µg of purified VLP protein were run of a 10% SDS gel and protein was detected by staining the gel using Coomassie Blue.

Ten cloned round 3 selectants were subjected to sequence analysis, which identified 8 different peptide sequences (Figure 1A). The peptide sequence SGIMPH was found in 3/10 selectants, while the other seven clones had unique sequences. When performing affinity selections on antibodies with linear epitopes, families of related sequences are often encountered. However, there was little primary sequence homology amongst the different peptides, with the exception of peptide 2 and peptide 3. Furthermore, none showed sequence identity to AIP4 (Figure 1A, right) suggesting that these peptides somehow structurally mimic AIP4, or that they bind an antibody paratope distinct from that occupied by AIP4.

The AP4-24H11 selectant VLPs were expressed in *E. coli* and purified by procedures we have described elsewhere [7]. Their elution behavior from the gel filtration matrix Sepharose CL-4B shows that each assembles into a particle the size expected of the VLP (not shown). Figure 1B shows the electrophoretic behavior of each VLP in agarose. Since only intact particles contain RNA, their staining with ethidium bromide verifies their intactness. Differences in electromobility are due mostly to charge differences imparted by the presence of the peptides on the VLP surface. To assess the purity of the VLP preparation prior to binding assays and immunizations, we analyzed protein content by SDS PAGE followed by Coommassie blue staining. VLPs (single chain dimer ∼28 kD) were effectively purified away from contaminating bacterial protein (Figure 1C).

**Figure 2:**
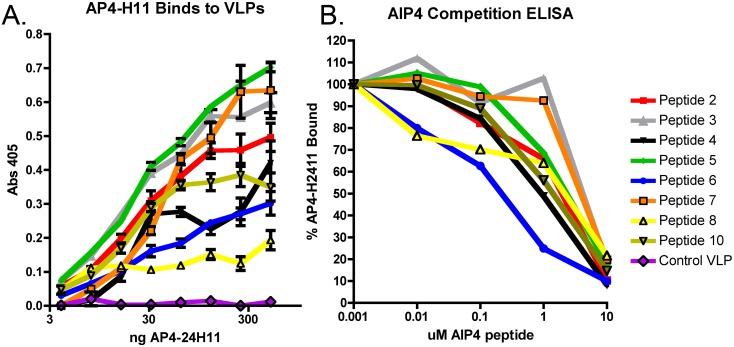
Affinity selected VLPs bind to and occupy the antigen binding site of mAb AP4-24H11. (A) ELISA was used to assess specific binding of VLP selectants to AP4-24H11. Wells were coated with the indicted VLPs and different concentrations of AP4-24H11 were applied. Error bars represent standard error of the mean. (B) For competition ELISAs, wells were coated with the indicated VLPs and AP4-24H11 was mixed with different concentration of the cyclical, bioactive AIP4 peptide prior to incubation with VLPs. Secondary antibody and detection was the same as above. As a control peptide, we used a linear form of AIP4. Results are representative of an experiment performed twice.

A direct ELISA was used to confirm that the affinity selected VLPs bound specifically to AP4-24H11. VLPs were used as the coating antigen (250 ng/well) and various amounts (0.1 to 500 ng) of AP4-24H11 were added to each well. AP4-24H11 bound to all of the VLPs displaying the selected peptides, whereas little to no binding of AP4-24H11 was observed with a control VLP (Figure 2A). The selected VLPs demonstrated a range of binding to AP4-24H11 with a ∼3.5-fold difference between the strongest binder (VLP displaying peptide 5) and the lowest (VLP displaying peptide 8). As an additional control, VLP coated wells were incubated with a mouse IgG control antibody using the same dilutions as used with the AIP4 mAb. Little or no binding was detected with the control antibody for any VLP samples (data not shown).

To ensure that selecting VLPs are binding to the antigen-binding site of AP4-24H11 rather than the Fc region, we investigated the ability of bioactive AIP4 peptide to compete with VLPs for antibody binding. VLPs were the coating antigen, and prior to the addition of AP4-24H11 (100 ng/well) various amounts of bioactive AIP4 peptide (10 µM–0.1 µM) were incubated with the mAb. The peptide/antibody mixture was added to the VLP coated wells and incubated for 1 hour. Similar to the results shown in Figure 2A, there was a range of peptide concentrations required to inhibit antibody binding (Figure 2B). Importantly, all VLPs were competed off the antibody by bioactive AIP4, suggesting that the selected VLPs are interacting with the antigen binding site of AP4-24H11.These data demonstrate that affinity selection can identify a population of VLPs displaying peptides that bind specifically to the mAb AP4-24H11.

**Figure 3:**
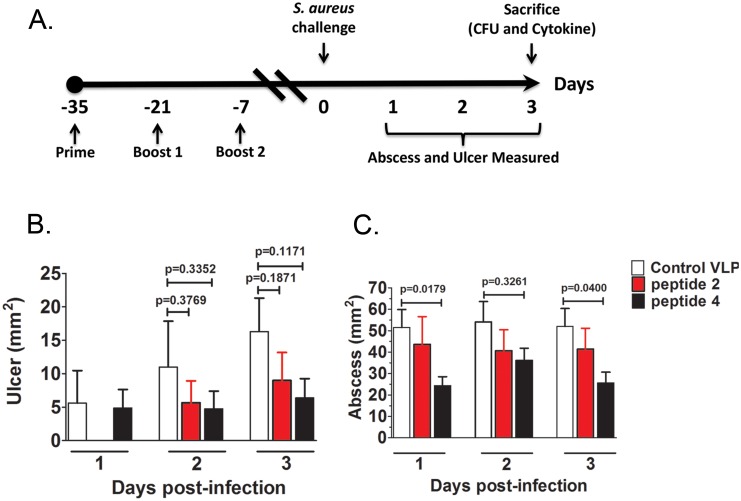
Vaccination with VLP mimotopes of AIP4 is efficacious in a mouse model of *S. aureus* SSTI. (A) Mice were vaccinated three times at two week intervals with 10 µg of VLP. One week after the final vaccination mice were inoculated subcutaneously with 1×10^8^ CFU of *S. aureus agr*-IV isolate AH1872, and abscess (B) and ulcer (C) areas were measured over the course of 72 hours. Data are shown as the mean ± SEM of at least two independent experments totalling 8–10 mice per group.

Next, we tested whether any of the selected VLPs could serve as an immunologic mimic of AIP4. Secreted virulence factors regulated by *agr*, such as Hla, mediate dermonecrosis, suggesting that vaccination with VLPs presenting immunologic mimics of the AP4-24H11 epitope would elicit protection against *agr*IV-mediated dermonecrosis. To test this, we vaccinated groups of 3 mice with VLPs presenting AP4-24H11-selected peptides or a VLP control, and challenged the mice by subcutaneous injection with *S. aureus agr*IV isolate AH1872 [24] (Figure 3A). We observed the mice for three days post-infection as we typically see maximum ulcer development by this time point followed by resolution over approximately the next seven days [31], [32]. Compared to VLP control vaccinated mice, mice vaccinated with peptide 4 VLPs showed significantly reduced abscess area on days 1 and 3 post-infection (Figure 3B). In addition, mice vaccinated with either peptide 2 or peptide 4 VLPs showed a trend toward reduced dermonecrosis (ulcer area) on days 2 and 3 post-infection, although this did not reach statistical significance (Figure 3C). Vaccination with VLPs displaying peptide 3 and peptides 5–10 were included in pilot testing but no protection was observed, therefore these VLPs were not included in further studies (data not shown).

**Figure 4:**
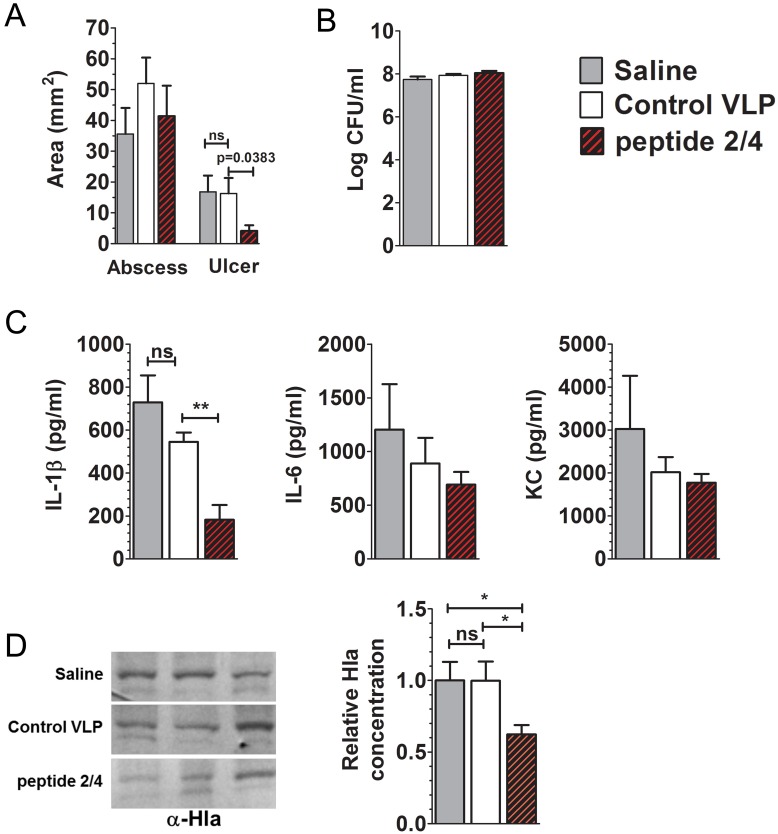
A combination vaccine of two VLP mimotopes limits pathogenesis in a mouse model of *S. aureus* dermonecrosis. Mice were vaccinated with 10 µg of a 1∶1 suspension of peptide 2 and peptide 4 and inoculated with 1×10^8^ CFU of AH1872 as described previously. At the apex of infection (day 3) (A) abscess and ulcer area were measured and (B) bacterial burden at the site of infection was determined, and (C) local cytokine and chemokine levels were determined. (D) Western blot showing relative HLA levels in tissue homogenate of vaccinated and challenged mice. Quantification based on Western blot band intensity relative to total protein concentration. Data are shown as the mean ± SEM of 6–10 mice per group. ns, not significant; *, p<0.05; **, p<0.01.

Based on the reduced ulcer area in mice vaccinated with peptide 2 or 4 VLPs, we asked whether vaccination with a combination of peptide 2 and peptide 4 VLPs (peptide 2/4-VLPs) would result in a significant reduction in dermonecrosis following *S. aureus* challenge. To address this, mice were vaccinated with a mixture (1∶1) of VLPs displaying peptide 2 and 4, control VLPs or PBS alone, and then challenged by subcutaneous injection with *S. aureus* AH1872. Whereas reductions in ulcer area in mice immunized with either peptide 2- or peptide 4-VLPs alone failed to reach statistical significance, mice immunized with combined peptide 2/4-VLPs had significantly reduced dermonecrosis compared to control vaccinated mice on day three post-infection (Figure 4A). Decreased ulcer area in peptide 2/4-VLP vaccinated mice was not due to a reduction in bacterial burden at the site of infection (Figure 4B) suggesting the decreased dermonecrosis resulted from inhibition of *agr* signaling. In support of this view, decreased dermonecrosis in the peptide 2/4-VLP vaccinated mice was associated with a significant decrease in local IL-1β levels, but not decreases in IL-6 or keratinocyte-derived chemokine (KC), compared to control vaccinated mice (Figure 4C). Such a decrease in local IL-1β levels is consistent with reduced *agr*-mediated expression of Hla, which causes pore-formation in host cells leading to NLRP3 inflammasome activation and IL-1β production [33]–[39]. To demonstrate that reduced IL-1β at the site of infection in peptide 2/4-VLP vaccinated mice was associated with decreased *agr*-mediated virulence factor expression, we measured Hla in tissue homogenate by Western blot analysis. As expected, peptide 2/4-VLP vaccinated mice had significantly less Hla at the site of infection compared to controls (Figure 4D). Together, these data suggest that peptides 2 and 4 identified by VLP affinity selection can serve as immunologic mimics of the *S. aureus* AIP4 mAb AP4-24H11 epitope and provide protection against *agr*-mediated dermonecrosis.

## Discussion

Recent technological advances have resulted in the isolation and characterization of a host of broadly neutralizing monoclonal antibodies having prophylactic and therapeutic effects against a variety of pathogens. We have developed a novel vaccine technology that takes advantage of these newly identified antibodies that allows for epitope discovery and mimicry on a highly immunogenic platform. We recently reported the use of this VLP selection platform to identify epitopes for several previously characterized mAbs that recognize linear epitopes [10]. In this paper, we extend these observations by identifying peptide mimics of the conformational epitope from the *S. aureus* AIP4 mAb AP4-24H11. Of critical importance is that compared to controls, co-immunization with two of the selected VLP candidates limited *agr*-signaling and pathogenesis during *S. aureus* SSTI as indicated by (1) decreased expression of the *agr-*regulated virulence factor Hla, (2) reduced local levels of the inflammatory cytokine IL-1 and (3) reduced dermonecrosis. Furthermore, although immunization did not impact bacterial burden at the time point evaluated, we and others have shown that, along with preventing or limiting dermonecrosis, disruption of *agr*-signaling or neutralization of Hla leads to increased bacterial clearance during resolution of infection [18], [31], [40]. This suggests the potential for vaccination with VLP-based AIP mimotopes to not only limit *agr*-dependent pathogenesis, but also to eventually contribute to host-mediated bacterial clearance. Importantly, these data provide proof-of-principle that our VLP technology provides a background upon which to develop efficacious vaccines against otherwise non-immunogenic, conformationally constrained epitopes. Antibodies are by nature polyspecific. In the universe of all possible short peptide sequences, an antibody may be capable of binding a number of them. Therefore, it is possible that only some affinity-selected peptides will bind the antigen-combining site through interactions mimicking those of the authentic antigen. It is well known, for example, that M13 phage display frequently finds so-called functional mimics, peptides that bind the antibody at paratopes distinct from the antigen itself. When utilized as immunogens, functional mimics fail to elicit antibodies with the desired specificity against the original antigen. We suspect functional mimics are especially readily encountered with antibodies like AP4-24H11 whose binding sites have not been optimized for binding to a simple linear peptide epitope. Immunogenic mimics, on the other hand, form molecular contacts with the selecting antibody similar to those that engage the antigen itself, and are therefore more likely to provoke antibodies that bind the antigen. Even in these cases, without detailed structural analysis it may be impossible to discern any obvious structural similarity between the original epitope and its affinity-selected immunogenic mimic. Of the 10 peptides we characterized here, 8 are apparently in the functional mimic category; they bind the antibody but fail to elicit antibodies with specificity for AIP4. However, two peptides elicited antibodies that served as immunogenic mimics as determined by their ability to provoke an immune response that protected against *S. aureus*-mediated dermonecrosis.

To date, no anti-*Staphylococcus aureus* vaccine has succeeded in Phase III clinical trials [41]. Such vaccines have primarily relied on immunization with *S. aureus* surface protein antigens, suggesting that strategies aimed at inducing opsonophagocytic antibodies are not sufficient to prevent disease by this pathogen. We are not alone in recognizing the possible utility of vaccines that target secreted virulence factors [42], [43]. For example, neutralization of the secreted virulence factor Hla using active vaccination or passive transfer of neutralizing antibodies has proven efficacious in several *S. aureus* infection models [20], [21], [38], [39], [44]–[46] and a vaccine targeting recombinant Hla was part of a recent clinical trial (NCT01011335). The previous example is one of several strategies based on inhibition of a single secreted virulence factor; however, immune-based approaches to inhibit *S. aureus* virulence factor expression on a global level have been limited (reviewed in [42]).

Park et al. recently demonstrated in vivo protection via passive administration of a mAb, directed against a synthetic AIP4 hapten, which prevents global virulence factor expression by inhibiting *agr* quorum sensing [15]. However, the availability of both prophylactic vaccines and therapeutic mAbs targeting *S. aureus agr*-regulated virulence would significantly increase the translational spectrum of this anti-staphylococcal virulence approach. Herein, we expanded on the work of Park et al. using MS2 VLP libraries and affinity selection to develop a vaccine strategy for *agr*-inhibition. Using this technique against the mAb AP4-24H11, we identified candidate vaccines with in vivo efficacy in a mouse model of *S. aureus* SSTI.

## Supporting Information

Figure S1 **VLP affinity selection to identify mimotopes of Mab AP4**-**24H11. Wells** of an ELISA plate were coated with the Mab AP4-24H11 and were incubated with VLP libraries displaying random peptides. RNA sequences from bound VLPs were recovered by RT-PCR and re-cloned into VLP expression constructs and VLP libraries enriched for peptides binding to AP4-24H11 were produced. Three rounds of biopanning were used and clones of the resulting VLPs were sequence for peptide identification and subsequent functional analysis.
(TIF)
