# Correction: Development of a Mimotope Vaccine Targeting the *Staphylococcus aureus* Quorum Sensing Pathway

**DOI:** 10.1371/journal.pone.0118160

**Published:** 2015-02-06

**Authors:** The PLOS ONE Staff

The sixth author, Pamela R. Hall, should not have been attributed equal contribution with John P. O’Rourke and Seth M. Daly.
