# Supplementary figures and images for: Development of a Mimotope Vaccine Targeting the Staphylococcus aureus Quorum Sensing Pathway
Source: PLoS One. 2014 Nov 7;9(11):e111198. doi: 10.1371/journal.pone.0111198 (PMC4224382; doi:10.1371/journal.pone.0111198)

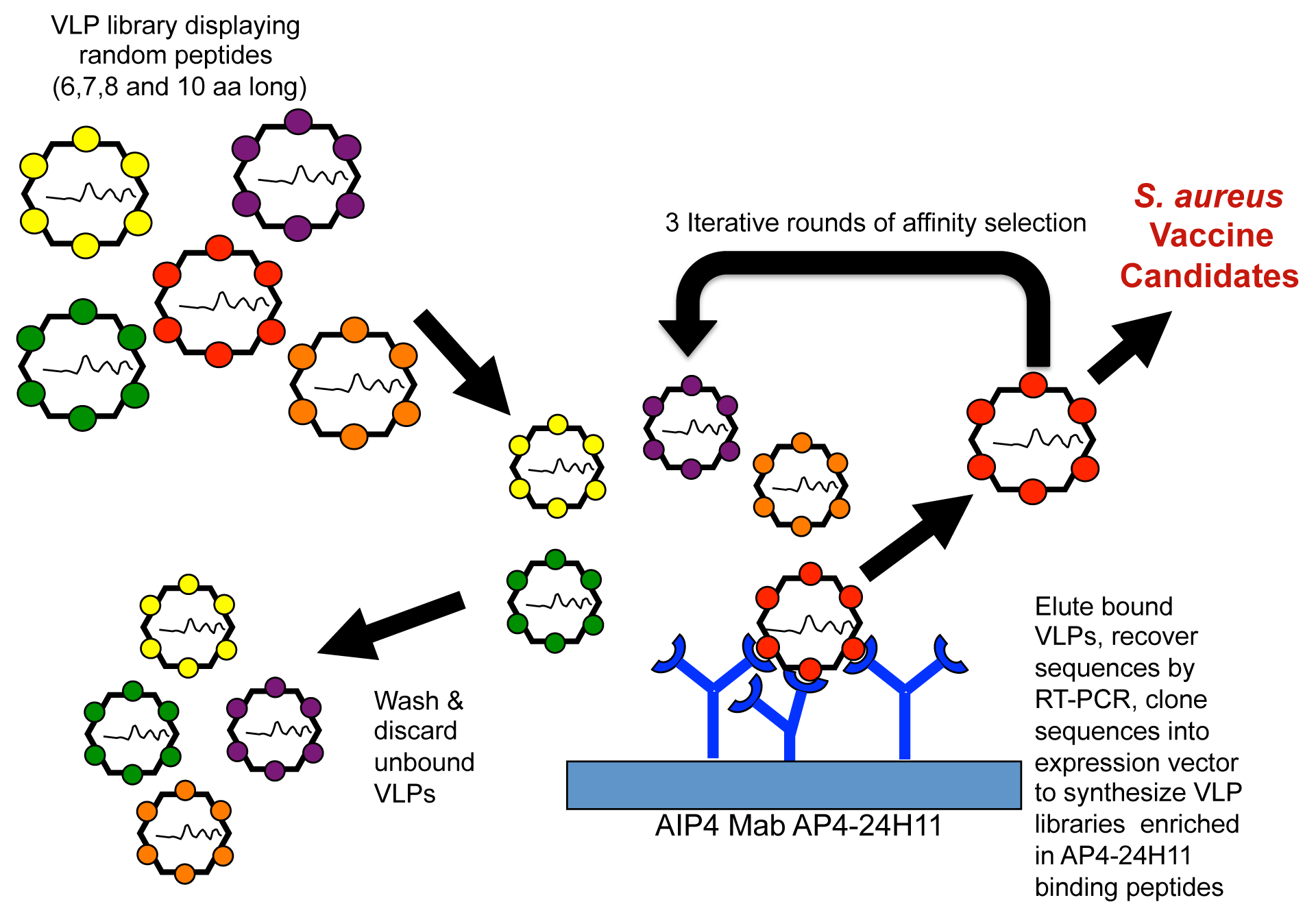

Supplement: Figure S1 — VLP affinity selection to identify mimotopes of Mab AP4-24H11. Wells of an ELISA plate were coated with the Mab AP4-24H11 and were incubated with VLP libraries displaying random peptides. RNA sequences from bound VLPs were recovered by RT-PCR and re-cloned into VLP expression constructs and VLP libraries enriched for peptides binding to AP4-24H11 were produced. Three rounds of biopanning were used and clones of the resulting VLPs were sequence for peptide identification and subsequent functional analysis. (TIF) [file pone.0111198.s001.tif]
